# Expansion of the Phenotype of Lymphatic Anomalies Caused by Somatic Activating BRAF Variant

**DOI:** 10.1002/pbc.32015

**Published:** 2025-08-30

**Authors:** Michael D. Fox, Sumukh Kumar, Allison D. Britt, Abhay S. Srinivasan, Lea F. Surrey, Seth E. Vatsky, Alexandra J. Borst, Hakon Hakonarson, Dong Li, Sarah E. Sheppard, Kristen M. Snyder, Denise M. Adams

**Affiliations:** 1 Division of Oncology, Children’s Hospital of Philadelphia, Philadelphia, Pennsylvania, USA; 2 Comprehensive Vascular Anomalies Program, Children’s Hospital of Philadelphia, Philadelphia, Pennsylvania, USA; 3 Perelman School of Medicine, University of Pennsylvania, Philadelphia, Pennsylvania, USA; 4 Division of Interventional Radiology, Children’s Hospital of Philadelphia, Philadelphia, Pennsylvania, USA; 5 Department of Pathology and Laboratory Medicine, Children’s Hospital of Philadelphia, Philadelphia, Pennsylvania, USA; 6 Division of Pediatric Hematology-Oncology, The University of North Carolina at Chapel Hill, Chapel Hill, North Carolina, USA; 7 Center of Applied Genomics, Children’s Hospital of Philadelphia, Philadelphia, Pennsylvania, USA; 8 Division of Pulmonary Medicine, Children’s Hospital of Philadelphia, Philadelphia, Pennsylvania, USA; 9 Unit on Vascular Malformations, Division of Intramural Research, Eunice Kennedy Shriver National Institute of Child Health and Human Development, Bethesda, Maryland, USA

**Keywords:** BRAF, central conducting lymphatic anomaly, complex lymphatic anomaly, generalized lymphatic anomaly, kaposiform lymphatic anomaly

## Abstract

**Background:**

The somatic activating variant in *BRAF* (p.V600E) was recently described as a novel cause of macrocystic head and neck lymphatic malformations in three individuals. Other recent studies profiling the genetic causes of more complex lymphatic anomalies identified this same pathogenic *BRAF* variant. Our aim was to expand the phenotypic description of the somatic *BRAF* p.V600E variant in individuals with vascular anomalies.

**Procedure:**

We searched the database of individuals with vascular anomalies at our institution for those identified as having complex lymphatic anomalies and somatic *BRAF* p.V600E variants. A comprehensive retrospective review of identified individuals’ electronic health records was conducted.

**Results:**

Six individuals with complex lymphatic anomalies had the *BRAF* p.V600E variant. All individuals had diffuse lymphatic malformations and abnormal lymphatic conduction. The conduction abnormalities were observed only after surgical interventions in five of the patients, in some cases, manifesting years later. There was immense phenotypic heterogeneity within the cohort.

**Conclusions:**

Complex lymphatic anomalies are an important new phenotype associated with the pathogenic BRAF p.V600E variant. Even in initially asymptomatic individuals within this patient population, longitudinal follow-up is necessary, and surgical intervention should be pursued with caution. Further investigation is needed to better understand the etiology of the conduction problems associated with this pathogenic variant to inform the multidisciplinary approach to treatment.

## 1 | Introduction

Complex lymphatic anomalies (CLA), which include generalized lymphatic anomaly (GLA), kaposiform lymphangiomatosis (KLA), Gorham Stout disease (GSD), and central conducting lymphatic anomaly (CCLA), are a highly morbid group of disorders characterized by abnormal development of lymphatic vessels resulting in diffuse lymphatic malformations (LM) and impaired lymphatic flow manifesting as effusions. Specific diagnosis of a CLA is made based on a combination of clinical presentation, imaging features, and histopathology (though often this is not available), but can be complicated by broad phenotypic heterogeneity as well as overlapping clinical features within this group of disorders [1–3].

GLA is notable for multifocal LM involving the soft tissues, thoracic and abdominal viscera and cavities, and bone. The bony involvement is often noncontiguous and does not result in destruction of the bone cortex. Affected tissue demonstrates dilated lymphatic channels that stain positive for D2-40 and PROX-1 (markers of lymphatic endothelium) [1–4]. KLA is similar to GLA in its areas of involvement, but commonly also involves coagulopathy with hemorrhagic effusions, and tends to have greater morbidity and higher risk of cardiopulmonary compromise due to its progressive and infiltrative nature. Individuals with KLA frequently have markedly elevated levels of angiopoietin-2, and histopathology demonstrates spindled lymphatic endothelial cells [1–5]. GSD, or “vanishing bone disease,” classically involves the bone in a contiguous pattern and results in progressive osteolysis with destruction of the bone cortex, but may also involve soft tissues and viscera. Evidence of this bony destruction on imaging is critical for the diagnosis, and histopathology demonstrates similar findings to GLA in addition to increased osteoclast activity [1–3]. CCLA is caused by dysfunction of or improper drainage into the thoracic duct and retrograde reflux of lymphatic fluid with leakage resulting in pleural or pericardial effusions, ascites, protein-losing enteropathy, or generalized edema. Lymphatic imaging is typically necessary to make this diagnosis [1–4].

Increased availability of somatic genetic testing has led to the identification of multiple pathogenic variants as causes of these disorders, though an unclear genotype–phenotype correlation remains [4, 6–15]. Identification of the genotype may help to classify these diagnoses more accurately and offer insight into optimal treatments. Therefore, further understanding of the molecular etiology of these diseases is critical.

The somatic activating variant *BRAF* c.1799T>A p.Val600Glu (V600E) was recently described as a novel cause of macrocystic head and neck LM in three individuals [16]. This variant had been previously reported to cause other vascular anomalies, but this was the first association of this gene with lymphatic anomalies [17–20]. Since then, this variant has been attributed to three cases of CCLA in publications by our institution [15, 21]. Here, we expand upon these studies to describe six cases of GLA, KLA, and/or CCLA associated with the *BRAF* variant, including the three individuals previously reported as well as three new individuals. These results expand the phenotypic description of this *BRAF* variant beyond isolated LMs to include CLAs.

## 2 | Methods

We conducted a single-center retrospective chart review at the Children’s Hospital of Philadelphia (CHOP) with all the data obtained as of February 2025. Inclusion criteria were patients with a confirmed *BRAF* V600E variant and a diagnosis of CLA. We queried the patient database at our institution for all patients with CLA and included those with a *BRAF* V600E variant. We have received an IRB exemption for this study. Genetic testing included the University of Pennsylvania Genetic Diagnostic Laboratory Somatic Overgrowth and Vascular Malformation (SOVM) Panel, CHOP’s Division of Genomic Diagnostics Solid Tumor Panel, and CHOP’s Center for Applied Genomics (CAG) research genetic panel [20].

## 3 | Case Descriptions

**FIGURE 1 |:**
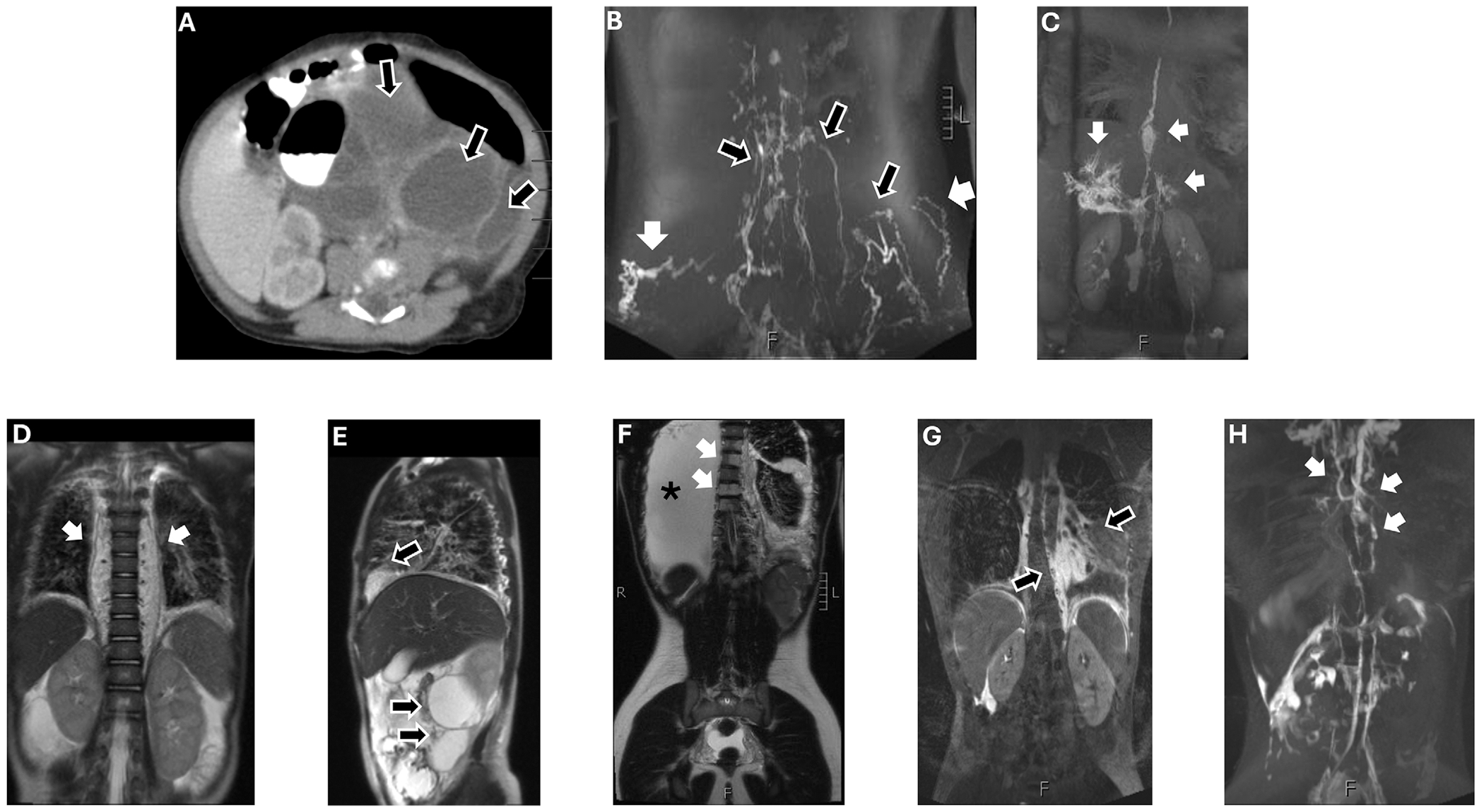
Imaging for Cases 1 and 2. (A–C) **Patient 1**: 18-year-old female with generalized lymphatic anomaly (GLA) and central conducting lymphatic anomaly (CCLA). (A) Computed tomography (CT) of a neonate showing multiple intra-abdominal cysts compatible with lymphatic malformation (black arrows). (B) Magnetic resonance lymphangiography (MRL) demonstrates dermal backflow from the groin (white arrows) with adjacent dilated lymphatic vessels in the superficial pelvis and gluteal region (black arrows). (C) MRL after hepatic injection shows an intact thoracic duct with multifocal saccular dilation of the lymphatic network in the retroperitoneum and cisterna chyli. (D–H) **Patient 2**: 16-year-old female with KLA. (D and E) Magnetic resonance imaging (MRI) of a toddler demonstrates extensive soft tissue prominence of the paravertebral soft tissues (white arrows), as well as cystic lesions in the paracolic gutters and lower chest (black arrows), consistent with multicompartmental lymphatic malformation. (F) MRI showing interval progression of disease with large right pleural effusion (asterisk) and bony involvement of the thoracic vertebrae (white arrows). (G) MRL after hepatic injection demonstrates proliferation and dilation of lymphatic channels in the chest (black arrows). (H) MRL after inguinal injection shows diffuse abnormal architecture of the thoracic lymphatics and collateral lymphatics with drainage to both the left and right venous angles (white arrows).

Patient #1 was diagnosed with an isolated LM of the mesentery and gluteal region on computed tomography (CT) imaging as a neonate (Figure 1A) and underwent resection of a large portion of the mesenteric mass and partial resection of the sigmoid colon at 11 days old, followed by colostomy closure at 4 months. She then had minimal follow-up until 13 years later, when she was incidentally found to have a left-sided chylothorax on scoliosis imaging. Dynamic contrast-enhanced magnetic resonance lymphangiography (DCMRL) demonstrated disconnected inguinal and central lymphatic system and dilated thoracic duct draining to collateral lymphatic network, extending through subcutaneous tissue and left paravertebral posterior intercostal lymphatic networks (Figure 1B,C). She underwent glue embolization of left-sided paravertebral and posterior intercostal lymphatic networks, and required bilateral lymphovenous anastomosis of the inguinal skin 1 year later due to weeping lymphatic blebs in the gluteal region. Pathology from a colon resection shows dilated submucosal lymphatic channels (Figure 4A,B) and irregular mesentery lymphatics. A biopsy of the gluteal skin shows mildly dilated and irregularly shaped lymphatics in the deep dermis (Figure 4C). Lymphatics were positive for D2-40 and PROX1. At this point, overall findings were consistent with a diagnosis of CCLA. Genetic testing from the biopsy also demonstrated a somatic *BRAF* V600E variant at 2.2%–2.6% variant allele frequency (VAF). She was treated with sirolimus for 3 years with no clinical improvement, then transitioned to trametinib for 5 months until dermatologic toxicities, mainly persistent acne, became severe with little improvement in her condition. She was then started on a BRAF inhibitor, vemurafenib, but discontinued after 2 months due to several toxicities, including nausea, weight loss, muscle aches, and hand contractures, coupled with a lack of improvement. Though she continues to have chronic pain and discomfort, her pleural effusion has resolved, and she is able to participate in activities of daily living despite no medical treatment for 2 years.

Patient #2, with a history of left inguinal hernia repair as a toddler, was incidentally diagnosed with “lymphangiomatosis” involving the mediastinum, spleen, and mesentery on magnetic resonance imaging (MRI) (Figure 1D,E) at age 3 as part of a workup for fever without a source. She was largely asymptomatic at the time and subsequently followed for 3 years before being lost to follow-up. At age 15, she presented with bacterial pneumonia complicated by bilateral bloody chylothorax. CT imaging showed a large left pleural effusion, and subsequent MRI confirmed extensive macrocystic and microcystic lymphatic malformations in the chest, abdomen, and pelvis with splenic involvement, perivascular thickening of central vessels, and confluent marrow replacement in the vertebral bodies (Figure 1F). She had normal coagulation studies, but a markedly elevated angiopoietin-2 level of 11,759 pg/mL in blood (reference range: 1434–4141 pg/mL). Research genetic testing of cell-free DNA (cfDNA) isolated from pleural lymphatic fluid revealed a somatic *BRAF* V600E variant at 0.2%–2.2% VAF [20]. Magnetic resonance lymphangiography (MRL) was performed after the genetic finding revealed abnormal lymphatic drainage into the mediastinum, chest, abdomen, and pelvis (Figure 1G,H). Additionally, biopsy of the right pleura showed irregular, dilated, thin-walled vascular spaces lined by CD31^+^ , D2-40^+^ , and PROX1^+^ endothelial cells, altogether consistent with lymphatics (Figure 4D–F). Features from radiology, clinical presentation, pathology, and genetics led to a diagnosis of KLA with central conduction abnormality. She was initially treated with sirolimus, changed to selumetinib once the *BRAF* variant was found, and then treated with dual therapy with sirolimus and selumetinib. However, she continued to have recurrent worsening of her chylothoraces requiring frequent thoracentesis and hospitalization. As such, she recently transitioned to dual therapy with dabrafenib and trametinib and underwent lung decortication and selective glue embolization. She has demonstrated substantial clinical improvement since this surgery and the dual therapy with dabrafenib and trametinib.

**FIGURE 2 |:**
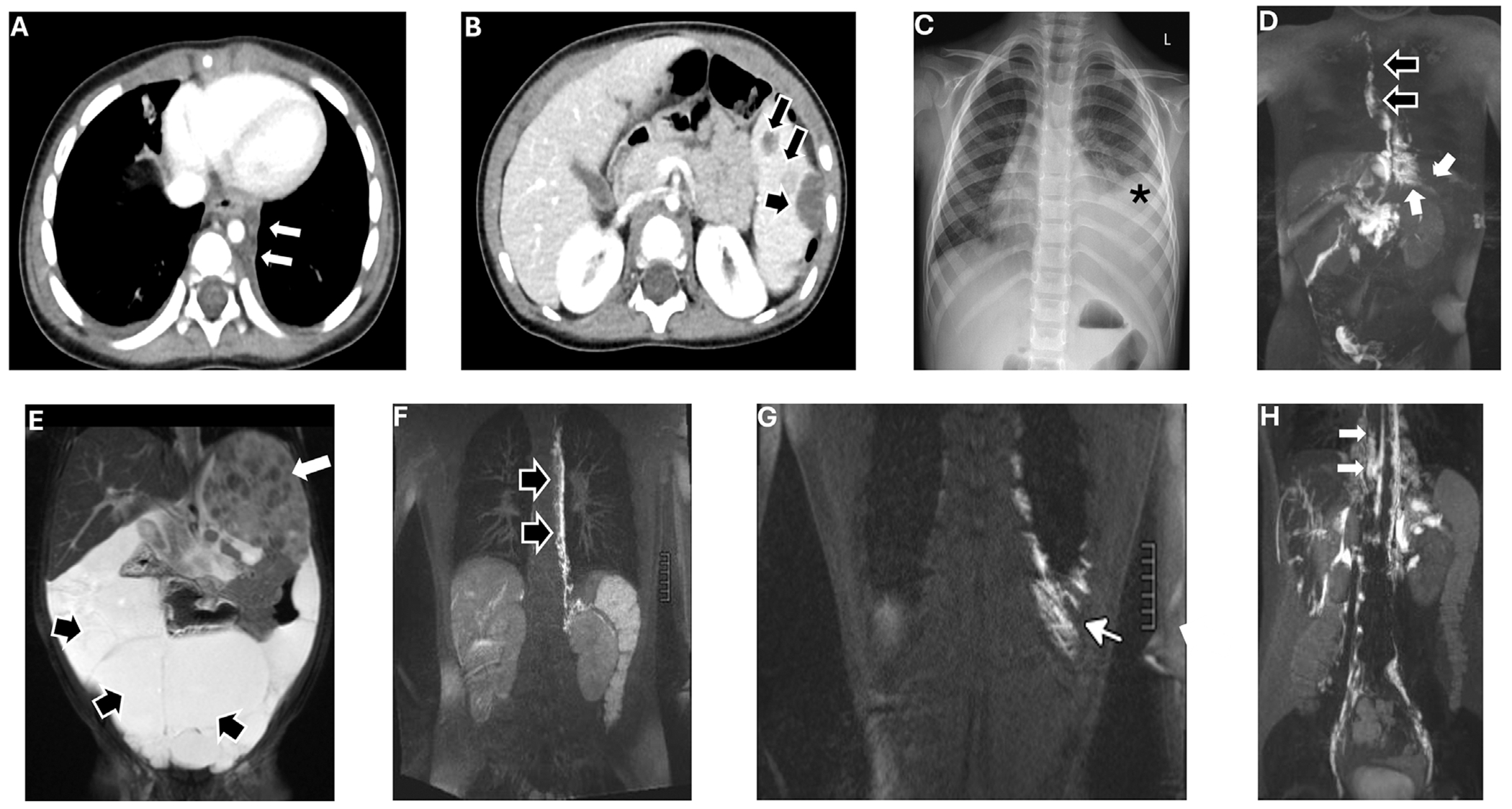
Imaging for Cases 3 and 4. (A–D) **Patient 3**: 4-year-old male with generalized lymphatic anomaly (GLA). (A and B) Computed tomography (CT) shows paraspinal lymphatic malformation (white arrows) and lymphatic cysts in the spleen (black arrows). (C) Chest radiograph demonstrates a large left pleural effusion (asterisk). (D) Magnetic resonance lymphangiography (MRL) after hepatic injection shows opacification of the paraspinal lymphatic malformation, with associated intercostal perfusion (white arrows), along with visualization of the right-sided thoracic duct (black arrows). (E–H) **Patient 4**: 21-year-old with GLA with abnormal lymphatic conduction. (E) Magnetic resonance imaging (MRI) of a toddler demonstrates multilocular lymphatic malformation of the abdomen (black arrows) with splenic involvement (white arrow). (F and G) MRL after liver injection shows collateral lymphatic channels along the left paravertebral network (black arrows) and intercostal perfusion (white arrow). (H) MRL with groin injection demonstrates dilated collateral lymphatic channels of the mediastinum (white arrows).

At 16 months, Patient #3 was incidentally found to have a well-circumscribed mass in the posterior mediastinum and numerous splenic lesions on CT imaging of the chest obtained after recurrent acute respiratory infections (Figure 2A,B). Biopsy of the chest mass demonstrated a combined venous (CD34^+^, D240^−^, PROX1 variable) and lymphatic (D240^+^) components with focal papillary endothelial proliferation. He developed chylothorax after the biopsy for which he required thoracentesis with chest tube placement; the effusion gradually improved with dietary modification and octreotide, but did not fully resolve. At 3 years old, he underwent DCMRL, which showed hepatopulmonary perfusion leaking into the left pleural space and a dilated and tortuous thoracic duct connecting the veins and mediastinal lymphatic malformation (Figure 2D), along with selective glue embolization. Overall findings were consistent with the diagnosis of GLA with central conduction abnormality. Research genetic testing of cfDNA isolated from thoracic duct lymphatic fluid and posterior mediastinal mass biopsy identified a somatic *BRAF* V600E variant at 0.4% and 1.3% VAF, respectively. He responded well to diuretics and the embolization procedure and did not require targeted pharmacotherapies. The patient has remained healthy without evidence of recurrent chylothorax or lymphatic conduction abnormality for nearly 3 years.

Patient #4 was diagnosed with “lymphangiomatosis” in the mesentery and spleen on MRI (Figure 2E) at age 3, at which time she underwent excision of an abdominal LM and splenectomy. Pathology from the LM showed abnormal thin-walled lymphatic channels filled with proteinaceous fluid and irregular mural smooth muscle that showed patchy D2-40 positivity with diffuse PROX1 and CD31 staining (Figure 4G–I). She remained in good health until age 16, when she presented with protein-losing enteropathy (PLE). Subsequent MRI showed cystic lesions in the chest and retroperitoneum as well as pleural effusions. DCMRL demonstrated cystic abdominal collections with intercostal and duodenal lymphatic perfusion (Figure 2F–H). Overall findings were consistent with a diagnosis of GLA with central conduction abnormality. Genetic testing of the abdominal tissue excised 13 years prior demonstrated a somatic *BRAF* V600E variant at 8% VAF. She responded well to diuretics and a low-fat, high-protein diet and did not require targeted pharmacotherapies.

**FIGURE 3 |:**
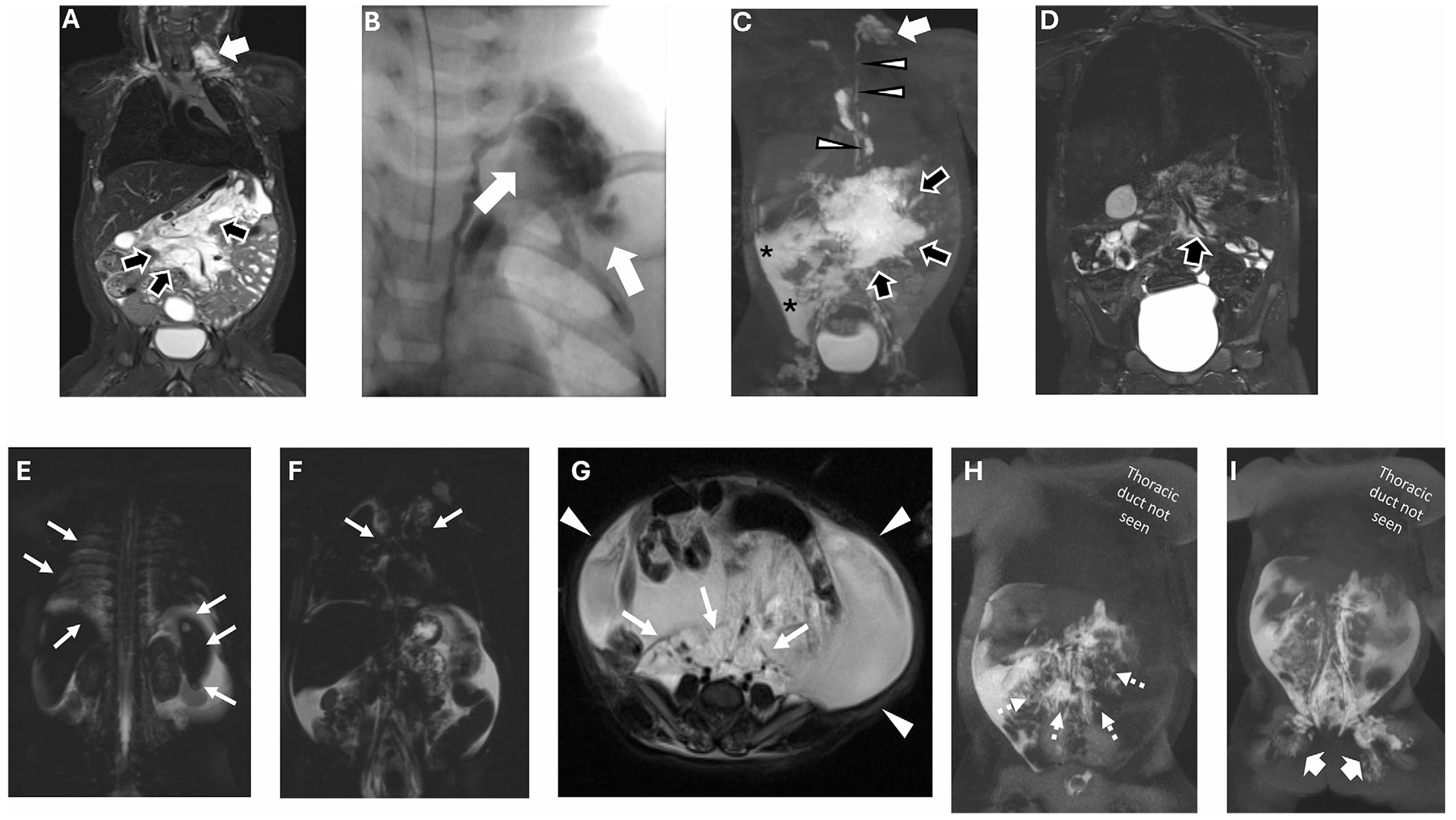
Imaging for Cases 5 and 6. (A–D) **Patient 5**: 4-year-old with generalized lymphatic anomaly (GLA) and central conducting lymphatic anomaly (CCLA). (A) Magnetic resonance imaging (MRI) during infancy shows lymphatic malformation centered in the mesentery (black arrows), with a smaller lymphatic malformation in the left supraclavicular region (white arrow). (B) Fluoroscopic imaging from direct lymphangiography after antegrade trans-abdominal access of the thoracic duct showing direct connection of the thoracic duct with the supraclavicular lymphatic malformation (white arrows). (C) Magnetic resonance lymphangiography (MRL) demonstrates the abdominal lymphatic malformation (black arrows) from liver and mesenteric injection with peritoneal leak (asterisks), as well as supraclavicular lymphatic malformation (white arrow) connected to the thoracic duct (white arrowheads) on inguinal injection. (D) Follow-up MRI at age 4 years after initiation of sirolimus showing a significant decrease in malformations (black arrow). (E–I) **Patient 6**: 14-month-old with GLA and lymphatic malformation (LM). (E–G) Axial and coronal T2-weighted images with fat suppression during infancy show infiltrative lymphatic malformation in the mediastinum, pleura, spleen, retroperitoneum, and pelvis (white arrows) and ascites (white arrowheads). (H and I) Maximum-intensity projection images of T1-weighted spoiled gradient echo images after injection of contrast into lymphatics show retrograde flow of contrast into mesentery and retroperitoneum (dashed arrow) after liver and mesenteric injection, but not into the thoracic duct. After groin injection, there is similar filling of retroperitoneal lymphatics and abnormal retrograde flow (thick arrows), but the thoracic duct still does not fill with contrast.

**FIGURE 4 |:**
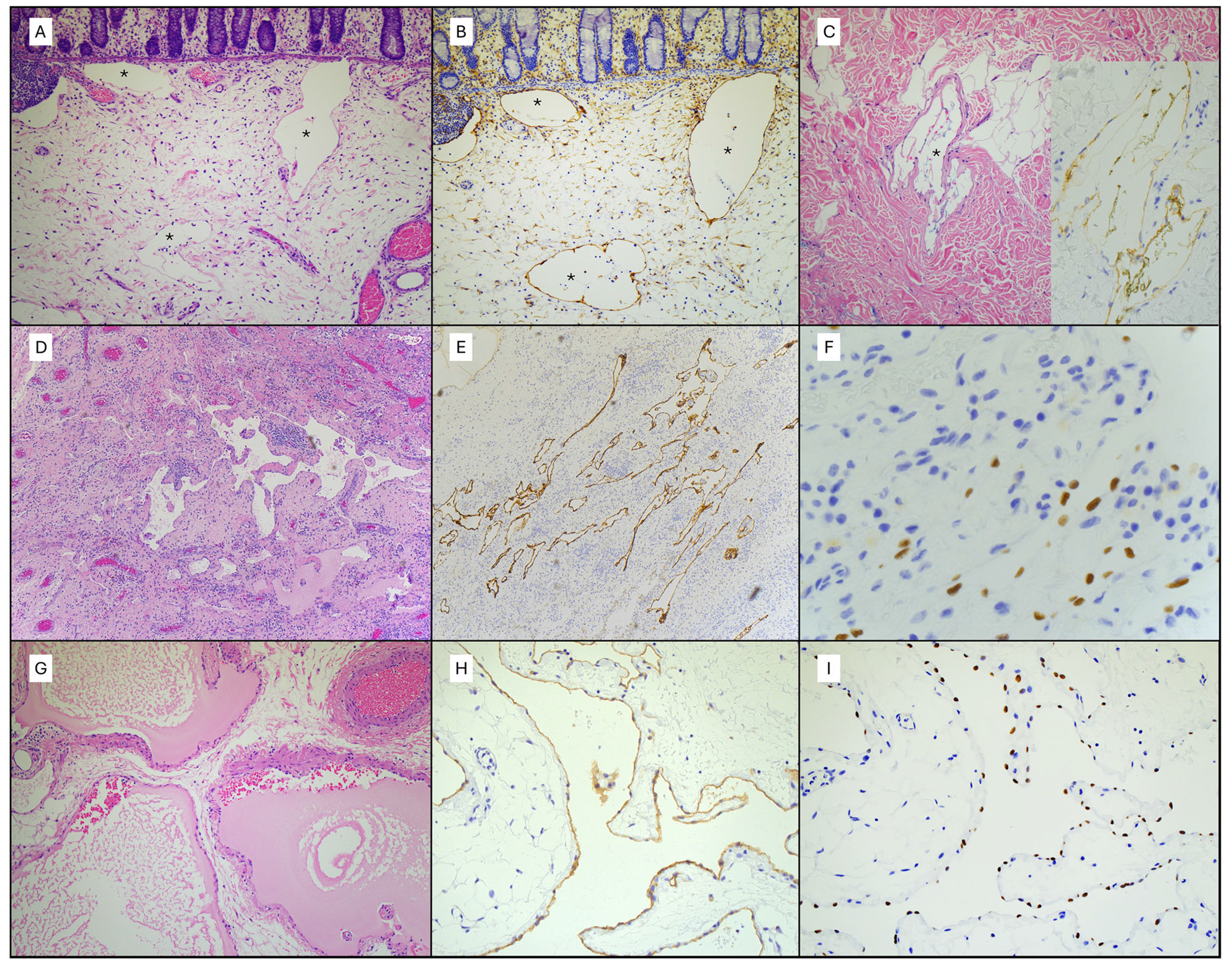
Pathology for Cases 1, 2, and 4. (A–C) Patient 1: The colon resection shows dilated submucosal lymphatics (*) (A, H&E, 100×), which are positive for D2-40 (B, 100×). Biopsy of gluteal skin shows irregular deep dermal lymphatics (C, H&E, 100×), which are D2-40 positive (C inset, 200×). (D–F) Patient 2: Pleural resection shows fibrosis, chronic inflammation, and a proliferation of irregular lymphatic channels (D, H&E, 50×), which are positive for D2-40 (E, 50×), PROX1 (F, 400×), and CD31 (not pictured). (G–I) Patient 4: Abdominal lymphatic malformation (LM) is notable for dilated thin-walled lymphovascular channels with irregular smooth muscle lining filled with proteinaceous material (G, H&E, 100×). The channels show D2-40 (H, 200×) and PROX1 (I, 200×) positivity, in addition to CD31 (not pictured).

Patient #5 had a prenatal ultrasound suspicious for intestinal atresia, but MRI at birth demonstrated diffuse LM in the neck and abdomen, consistent with GLA. She was started on sirolimus at 2 months of age and initially did well on this medication until she developed PLE and anasarca at 16 months of age. DCMRL demonstrated mesenteric LM with bowel wall involvement as well as impaired communication between the mesenteric and central lymphatic systems (Figure 3A–D). At this point, the diagnosis evolved to that of GLA with central conduction abnormality. Genetic testing of cfDNA from pleural lymphatic fluid demonstrated a somatic *BRAF* V600E variant at 0.2% VAF. She has responded well to treatment with sirolimus and a low-fat diet.

Patient #6 was prenatally diagnosed with hydrops and small bowel obstruction and born prematurely at 29 weeks of gestational age. She underwent exploratory laparotomy with repair of gastric perforation on day of life 5. MRI demonstrated large volume ascites and diffuse LM involving the chest and inguinal areas, and DCMRL showed no filling of the duct after hepatic, mesenteric, or inguinal injections, indicative of primary lymphatic flow disorder (Figure 3E–I). Overall findings were consistent with GLA with central conduction abnormality. Research genetic testing of cfDNA from plasma and thoracic duct lymphatic fluid demonstrated a somatic *BRAF* V600E variant at 0.5% and 2.0% VAF, respectively. She is followed at an outside institution, with no further data available for review.

## 4 | Discussion

This report describes six cases of CLAs associated with the pathogenic *BRAF* V600E variant in the absence of other known pathogenic variants. Though all six individuals had diffuse LMs and abnormal lymphatic conduction, the cases demonstrate the phenotypic heterogeneity associated with this variant and further illustrate the spectrum of morbidity associated with these disorders. To our knowledge, this is the first detailed association of the *BRAF* V600E with CLAs and expands the phenotypic description of this variant in patients with vascular anomalies [16–20].

The individuals described in this study underwent varied treatments, ranging from observation without intervention to dietary modification to targeted pharmacotherapies to surgical and procedural interventions. This study did not evaluate the efficacy of specific treatments in this patient population. However, our experience suggests that elucidating the genetic basis for CLAs may inform a more directed approach to treatment, especially as it relates to targeted pharmacotherapy, though additional studies are needed.

Interestingly, five of the six individuals reported in our case series developed symptoms related to abnormal lymphatic conduction only after surgical interventions. Additionally, in the majority of cases, the manifestation of this abnormal lymphatic conduction was found years after their initial diagnoses and surgeries. We are limited in our ability to determine whether these interventions may have disrupted the underlying lymphatic anatomy and caused or contributed to this abnormal conduction, or if the abnormal lymphatic conduction was an inherent aspect of these individuals’ CLA phenotypes that would develop over time. Regardless, we propose added caution when considering procedural or surgical interventions in patients with CLAs, especially in the setting of a known causative *BRAF* V600E variant. We also emphasize the importance of regular longitudinal follow-up even for those individuals who are seemingly asymptomatic without treatment.

CLAs are a group of diseases that can be challenging to diagnose given significant overlap in clinical presentation, imaging, and histopathology. As we develop further understanding of the molecular etiology of these disorders, it is apparent that there may be significant genetic overlap as well. Identification of the causative gene variant in an individual diagnosed with a CLA may be critical to optimizing that individual’s unique treatment plan.

## Abbreviations:

CAG: Center for Applied Genomics
CCLA: central conducting lymphatic anomaly
CHOP: Children’s Hospital of Philadelphia
CLA: complex lymphatic anomaly
CT: computed tomography
DCMRL: dynamic contrast-enhanced magnetic resonance lymphangiography
GLA: generalized lymphatic anomaly
GSD: Gorham Stout disease
KLA: kaposiform lymphangiomatosis
LM: lymphatic malformation
MRI: magnetic resonance imaging
PLE: protein-losing enteropathy
SOVM: Somatic Overgrowth and Vascular Malformation Panel
VAF: variant allele frequency
